# Predicting the Evolution of Sexual Dimorphism in Gene Expression

**DOI:** 10.1093/molbev/msaa329

**Published:** 2021-01-29

**Authors:** David Houle, Changde Cheng

**Affiliations:** 1 Department of Biological Science, Florida State University, Tallahassee, FL, USA; 2 Department of Integrative Biology, University of Texas, Austin, TX, USA

**Keywords:** gene expression, sexual dimorphism, G matrix, genetic constraint, sex-biased gene expression

## Abstract

Sexual dimorphism in gene expression is likely to be the underlying source of dimorphism in a variety of traits. Many analyses implicitly make the assumption that dimorphism only evolves when selection favors different phenotypes in the two sexes, although theory makes clear that it can also evolve as an indirect response to other kinds of selection. Furthermore, previous analyses consider the evolution of a single transcript or trait at a time, ignoring the genetic covariance with other transcripts and traits. We first show which aspects of the genetic-variance–covariance matrix, **G**, affect dimorphism when these assumptions about selection are relaxed. We then reanalyze gene expression data from *Drosophila melanogaster* with these predictions in mind. Dimorphism of gene expression for individual transcripts shows the signature of both direct selection for dimorphism and indirect responses to selection. To account for the effect of measurement error on evolutionary predictions, we estimated a **G** matrix for eight linear combinations of expression traits. Sex-specific genetic variances in female- and male-biased transcription, as well as one relatively unbiased combination, were quite unequal, ensuring that most forms of selection on these traits will have large effects on dimorphism. Predictions of response to selection based on the whole **G** matrix showed that sexually concordant and antagonistic selection are equally capable of changing sexual dimorphism. In addition, the indirect responses of dimorphism due to cross-trait covariances were quite substantial. The assumption that sexual dimorphism in transcription is an adaptation could be incorrect in many specific cases.

## Introduction

The prevailing model of the evolution of sexual dimorphism (e.g., Rice and Chippindale 2008; Bonduriansky and Chenoweth 2009; Cox and Calsbeek 2009) supposes that a sexually monomorphic ancestral population is subjected to selection that drives the male and female means apart. Examples of extreme sexual dimorphism tied to sex-specific functions, such as the horns of bighorn sheep, constitute intuitive evidence for this scenario. Another kind of evidence for this model is the existence of sexual conflict, that is, persistent antagonistic selection on sex-specific traits (Rice 1984; Partridge and Hurst 1998; Arnqvist and Rowe 2005). Since the genomes of the two sexes are similar, differing principally in the sex chromosomes, a population may evolve toward the sex-specific optima very slowly (Lande 1980), resulting in sexual dimorphism that is less than optimal. There is direct experimental evidence in favor of such conflicts from experiments that alter the relative strength of selection on the sexes, and result in changes in traits expressed in both sexes (Prasad et al. 2007). We call this the genomic constraint hypothesis of sexual dimorphism.

A readily available source of high-throughput data on sexual dimorphism is messenger RNA abundance in the sexes (Ingleby et al. 2015; Mank 2017). Furthermore, differences in gene expression are likely to underlie dimorphism in many other phenotypic traits. Several lines of evidence suggest that genomic constraint and intralocus conflict shapes the evolution of gene expression in *Drosophila melanogaster.*Hollis et al. (2014) minimized sexual conflict by enforcing monogamous mating for more than 100 generations, and observed that expression of sex-biased transcripts shifted in the direction of female expression, suggesting that biased genes are on an average less different in their expression than would be optimal. Griffin et al. (2013) reanalyzed the gene expression data of Ayroles et al. (2009), and found that estimates of genetic correlations between male and female gene expression for a particular gene, *r*_MF_, were correlated with multiple aspects of sexual dimorphism, including the degree of sex bias within *D. melanogaster*, the rate of evolution of expression bias among species, and the degree of sexually antagonistic selection (SAS) that *D. melanogaster* experiences. In addition, Innocenti and Morrow (2010) showed that transcripts exhibiting sex by fitness interactions, which may indicate antagonistic selection, have larger *r*_MF_ than other transcripts. All of these results are expected under the genomic constraint hypothesis.

On the other hand, concordant selection on the sexes can also result in increases in dimorphism if the sexes differ in their evolvability (Fisher 1930; Lande 1980; Leutenegger and Cheverud 1982; Cheverud et al. 1985; Lynch and Walsh 1998, chapter 24; Bonduriansky and Chenoweth 2009; Wyman et al. 2013). The model of Connallon and Clark (2014) elegantly combines the effects of antagonistic and concordant selection. It shows that under general conditions almost any change in the sex-specific optima will generate at least transient dimorphism and sexual conflict even when selection on the two sexes is initially concordant. Despite the widespread acknowledgment by theoreticians of concordant selection’s possible role in the evolution of dimorphism, analyses of empirical data rarely incorporate this possibility. The analysis of gene expression by Griffin et al. (2013) did not consider any alternatives to the genomic constraint hypothesis. We have recently proposed a transformation of the **G** matrix that separates the genetic variation allowing responses to concordant versus antagonistic selection (Cheng and Houle 2020), and makes clear that the conditions under which concordant selection can have large effects on sexual dimorphism are not rare. In addition, the sex-averaged expression of genes in the genus *Drosophila* is, on average, subject to very weak stabilizing selection that allows a substantial short-term role for genetic drift (Bedford and Hartl 2009).

A quantitative genetic framework is useful to capture the ability of genetic variation to either allow or constrain the evolution of sexual dimorphism (Lande 1980). The additive variances and covariances among male- and female-expressed traits, summarized in a **G** matrix, make it possible to predict how traits will respond to current selection. The covariances between trait values in one sex with those in the other sex are key to the potential resolution of sexual conflicts. These covariances can be collected into a submatrix of **G** known as the **B** matrix. The diagonals of the **B** matrix are the genetic covariances of homologous traits expressed in different sexes, and are commonly summarized using the genetic correlation $r_{MF}$.

We set out to expand on the analyses of Griffin et al. (2013) because they made simplifying assumptions about the genetic context in which dimorphism evolves, and their analysis does not fully match the nature of the data. As noted above, $r_{MF}$ values are not the only aspects of **G** that affect the evolution of sexual dimorphism. Cheng and Houle (2020) show precisely which other parts of the **G** matrix affect the evolution of dimorphism under natural selection. These additional aspects of **G** may be particularly accessible to study in the case of gene expression. Each transcript genetically covaries with a vast array of other transcripts, as well as other kinds of phenotypes. Indeed, Ayroles et al. (2009), the workers who collected the data reanalyzed by Griffin et al., detected 241 clusters of transcripts that were positively correlated with transcripts in that cluster, and more independent of, or even negatively correlated with expression of transcripts in other clusters. These covariances may cause a focal trait and its dimorphism to evolve as an indirect response to selection (Blows and Hoffmann 2005; Hansen and Houle 2008; Walsh and Blows 2009). The relationships between *r*_MF_ and dimorphism that Griffin et al. (2013) and others have observed implies that contemporary **G** matrices do reflect genetic variation responsible for sexual dimorphism over long periods of evolution, despite the many theoretical reasons that **G** matrices can change in the short term. Furthermore, a growing number of studies has also found that the properties of a **G** matrix predict patterns of evolution in other contexts as well (Bolstad et al. 2014; Houle et al. 2017).

Our analysis of the Ayroles et al. (2009)*D. melanogaster* expression data set allows us to explore the possible importance of concordant and antagonistic selection on sexual dimorphism from two rather different perspectives. First, we can ask what aspects of the current pattern of variances and covariances are correlated with the current level of sexual dimorphism. These correlations reflect the influence of past selection. Second, we can predict the effects of current selection on dimorphism in gene expression.

To detect the signature of past selection, we analyzed the sample covariance matrix of sex-specific line means, which we term G*, to see if cross-transcript and cross-sex covariances can explain more variation in sexual dimorphism than $r_{MF}^{*}$ alone. Although this approach is useful for exploratory purposes, only 40 genotypes were measured in the Ayroles et al. (2009) study, rendering any statistical tests using this approach invalid.

To predict how current selection will shape dimorphism, we estimated a **G** matrix for a small number of linear combinations of gene expression traits that collectively have statistically significant genetic variances. This yields a statistically unbiased **G** matrix that we use to compare the predicted selection responses to antagonistic and concordant selection.

Before proceeding to these results, we first use some simple models of the response of sexual dimorphism to selection to build intuition about the roles of asymmetries in male and female genetic variance, and of cross-trait covariances in the evolution of sexual dimorphism. The major take-home lesson of this theoretical section is that the magnitude of indirect responses to SCS can rival the direct responses to SAS that are traditionally regarded as responsible for sexual dimorphism. Readers who wish to bypass the mathematical development can skip ahead to the empirical results and the worked examples of responses to selection in the Results section.

## What Influences the Rate of Evolution of Sexual Dimorphism?

In this section, we show which parts of the **G** matrix influence the evolution of sexual dimorphism under two extreme selective scenarios for one and two traits. The first scenario is symmetrical sexually antagonistic selection (SAS) where male and female traits are selected to change in opposite directions, and dimorphism is thus under direct selection. The second scenario is symmetrical sexually concordant selection (SCS) where male and female traits are selected in the same direction. Dimorphism can also evolve in this scenario as an indirect response when male and female traits respond at different rates.

Consider *k* quantitative traits, with phenotypic values *z_1_, z_2_, …, z_k_*. Lande (1980) formulated the quantitative genetic prediction equation:

(1) $$\Delta \bar{z}=G\beta$$

that predicts a *k *×* *1 vector of predicted responses to selection, $\Delta \bar{z}$, from a *k *×* *1 vector of partial regression coefficients of fitness on trait, β, and the *k *×* k* additive genetic covariance matrix, **G**. Lande (1980) then generalized this to consider the same *k* traits separately in each sex:

(2) $$\left[\begin{array}{c} \Delta {\bar{z}}_{M}\\ \Delta {\bar{z}}_{F} \end{array}\right]=\frac{1}{2}\left[\begin{array}{cc} G_{M} & B\\ B^{T} & G_{F} \end{array}\right]\left[\begin{array}{c} \beta_{M}\\ \beta_{F} \end{array}\right],$$

where *F* and *M* index female and male response vectors, **G** matrices, and selection gradient vectors. Individual elements of the **G**_*M*_ and **G**_*F*_ matrices will be written $m_{ij}$ and $f_{ij}$, respectively. The *k *×* k* matrix **B** contains the covariances between traits expressed in the other sex, with $b_{ij}$ denoting the covariance of the *i*th trait in females with the *j*th trait in males. The diagonal of **B** is the covariance of homologous traits between the sexes, whereas the off-diagonal elements are the covariances between sexes among nonhomologous traits. The **B** matrix is not necessarily symmetric, as the alleles may have different magnitudes of effects on each sex.

### One Trait Case

Griffin et al.’s (2013) analysis focused on $r_{MF}$ as an indicator of the degree of constraint. Consider the simple case of SAS on the expression of one gene. The predicted response to selection is:

$$\Delta \bar{z}=\left[\begin{array}{c} \Delta {\bar{z}}_{M}\\ \Delta {\bar{z}}_{F} \end{array}\right]=\frac{1}{2}\left[\begin{array}{cc} m & b\\ b & f \end{array}\right]\left[\begin{array}{c} \beta \\ -\beta \end{array}\right]=\frac{\beta }{2}\left[\begin{array}{c} m-b\\ b-f \end{array}\right].$$

The change in sexual dimorphism under antagonistic selection is:

$$\begin{array}{c} \Delta_{a}=\Delta {\bar{z}}_{M}-\Delta {\bar{z}}_{F}=\frac{\beta }{2}\left(m+f-2b\right)\\ =\beta \left(\bar{g}-b\right) \end{array},$$

where $\bar{g}=\left(m+f\right)/2$ is the average genetic variance in the two sexes. The quantity $\left(\bar{g}-b\right)$ is the genetic variance for sexual dimorphism.

The effect of $r_{MF}$ can be highlighted by substituting $b=r_{MF}\sqrt{mf}$, which yields,

(3) $$\Delta_{a}=\beta \bar{g}\left(1-d\;r_{MF}\right),$$

where

$$d=\frac{\sqrt{mf}}{\bar{g}},$$

the ratio of the geometric and arithmetic means of the male and female variances. This has a maximum value of *d *=* *1 when the ratio of male to female variances is 1. A positive $r_{MF}$ restricts the rate of change in sexual dimorphism, whereas a negative correlation increases it. The sign of the change in dimorphism is controlled by the sign of *β*, as both of the other terms must be positive. Reviews of empirical estimates of $r_{MF}$ show that they are positive on an average (Poissant et al. 2010; Griffin et al. 2013). In the special case when *m = f*, we recover the familiar result that:

$$\Delta_{A}=\beta \bar{g}\left(1-r_{MF}\right),$$

the starting point for Griffin et al.’s analysis. This is, however, also the state where the influence of $r_{MF}$ is maximized, as *d *<* *1 whenever m≠f. This effect is relatively small when the ratio of *m* to *f* is fairly close to 1; for example, when the ratio is 2:1 or 1:2, *d* is 0.94; *d* is not halved until the ratio of variances is nearly 14:1.

Under concordant selection, the change in dimorphism is:

(4) $$\Delta_{C}=\frac{\beta }{2}\left(m-f\right).$$

This can be put in the same form as equation (3), yielding:

(5) $$\Delta_{C}=\beta \bar{g}\sqrt{1-d^{2}}.$$

Thus, concordant selection will change dimorphism whenever there are different genetic variances in the two sexes, as previously noted by many authors (see the Introduction). The response can either increase or decrease sexual dimorphism if selection is on a previously dimorphic trait.

Comparison of equations (3) and (5) shows that in the single trait case, the rate of change in dimorphism will be higher under concordant selection than antagonistic selection of equal strength, when $d<\left(2r_{MF}\right)/\left(r_{MF}^{2}+1\right)$. This condition is increasingly easy to satisfy the larger $r_{MF}$is. At the average value $r_{MF}=0.75$ found in Poissant et al.’s (2010) review of intersexual correlations, the condition is met when the ratio of the larger of *m* and *f* to the smaller is 1.5 or larger; when $r_{MF}=0.9$ a ratio of 1.16 is enough for concordant selection to dominate changes in dimorphism.

### Two Trait Case

When *k *=* *2, differences in genetic variances between the sexes remain a key source of dimorphism. However, to focus on other features of the **G** matrix that can promote dimorphism, but are absent from the *k *=* *1 case, we consider the special case when all trait variances are 1, yielding the **G** matrix:

$$G=\left[\begin{array}{cccc} 1 & r_{M1M2} & r_{\text{MF}1} & r_{M2F1}\\ r_{M1M2} & 1 & r_{M1F2} & r_{\text{MF}2}\\ r_{\text{MF}1} & r_{M1F2} & 1 & r_{F1F2}\\ r_{M2F1} & r_{\text{MF}2} & r_{F1F2} & 1 \end{array}\right]$$

If only the focal trait is under selection (e.g., for antagonistic selection $\beta_{A}^{T}=\left[\begin{array}{cccc} \beta & 0 & -\beta & 0 \end{array}\right]$), the above results hold for the selected trait. However, there is also an indirect response, which leads to a change in dimorphism in $z_{2}$ under antagonistic selection on $z_{1}$ of:

$$\Delta_{A\cdot 2}=\beta \;\left(\left[\frac{r_{M1M2}+r_{F1F2}}{2}\right]-\left[\frac{r_{M1F2}+r_{M2F1}}{2}\right]\right).$$

The first term in brackets is the average correlation between the traits in males and females, and the second is the average cross-sex correlation. Under concordant selection, sexual dimorphism changes at the rate:

$$\Delta_{C\cdot 2}=\beta \;\left(\left[\frac{r_{M1M2}-r_{F1F2}}{2}\right]+\left[\frac{r_{M1F2}-r_{M2F1}}{2}\right]\right),$$

which is affected by the asymmetry of the cross-trait correlations between **G**_*M*_ and **G**_*F*_ (the first term in brackets), and between the off-diagonal elements of **B** (the second bracketed term).

The fact that there are indirect responses to selection makes the interpretation of the existing degree of dimorphism in particular traits more challenging. For example, it is quite possible for the change in dimorphism of the selected trait to be less than that of the unselected trait. The direct response to antagonistic selection, $\Delta_{A.1}$, will be small when $r_{MF1}\approx 1$, making it plausible that $\Delta_{A.2}>\Delta_{A.1}$ if within-sex, cross trait correlations$r_{M1M2}\;\text{and}\;r_{F1F2}$ are larger than cross-sex cross-trait correlations $r_{M1F2}\;\text{and}\;r_{M2F1}$.

If both traits are under directional selection, there are two orthogonal, SAS vectors $\beta_{A1}^{T}=\left[\begin{array}{cccc} \beta & \beta & -\beta & -\beta \end{array}\right]$ and $\beta_{A2}^{T}=\left[\begin{array}{cccc} \beta & -\beta & \beta & -\beta \end{array}\right]$. Vector $\beta_{A1}$, for example, predicts a response vector:

$$\Delta {\bar{z}}_{A1}=\frac{\beta }{2}\left[\begin{array}{c} 1-r_{MF1}+r_{M1M2}-r_{M1F2}\\ 1-r_{MF2}+r_{M1M2}-r_{M2F1}\\ -1+r_{MF1}-r_{F1F2}+r_{M2F1}\\ -1+r_{MF2}-r_{F1F2}+r_{M1F2} \end{array}\right]$$

and a change in dimorphism of:

(6) $$\begin{array}{c} \Delta_{A1}=\beta \left[\begin{array}{c} \left(1-r_{MF1}\right)+\left(\frac{r_{M1M2}+r_{F1F2}}{2}\right)-\left(\frac{r_{M2F1}+r_{M1F2}}{2}\right)\\ \left(1-r_{MF2}\right)+\left(\frac{r_{M1M2}+r_{F1F2}}{2}\right)-\left(\frac{r_{M2F1}+r_{M1F2}}{2}\right) \end{array}\right]\\ =\beta \left[\begin{array}{c} 1-r_{MF1}+{\bar{r}}_{w}-{\bar{r}}_{b}\\ 1-r_{MF2}+{\bar{r}}_{w}-{\bar{r}}_{b} \end{array}\right]. \end{array}$$

The direct responses are given by the leftmost term in parentheses. The middle term gives the indirect responses due to the average within-sex correlations between traits, ${\bar{r}}_{w}$. The final term gives the indirect responses due to the average off-diagonal (between-sex) correlations in **B,**${\bar{r}}_{b}$. The complementary antagonistic selection gradient, $\beta_{A2}$, reverses the signs of the indirect effects, so that ${\bar{r}}_{w}$retards the evolution of dimorphism, whereas ${\bar{r}}_{b}$ facilitates it. Thus, the effects of ${\bar{r}}_{w}$ and ${\bar{r}}_{b}$ are always antagonistic to each other. Dimorphism is always constrained by $r_{MF}$.

Concordant selection, $\beta_{C1}^{T}=\left[\begin{array}{cccc} \beta & \beta & \beta & \beta \end{array}\right]$, predicts a change in dimorphism of:

(7) $$\begin{array}{c} \Delta_{C1}=\frac{\beta }{2}\left[\begin{array}{c} \left(r_{M1M2}-r_{F1F2}\right)+\left(r_{M1F2}-r_{M2F1}\right)\\ \left(r_{M1M2}-r_{F1F2}\right)+\left(r_{M2F1}-r_{M1F2}\right) \end{array}\right]\\ =\frac{\beta }{2}\left[\begin{array}{c} r_{\delta w}+r_{\delta b}\\ r_{\delta w}+r_{\delta b} \end{array}\right]. \end{array}$$

In this special case, where all genetic variances are equal, there is no direct response in dimorphism. The first term inside the parentheses, $r_{\delta w}$, is the indirect response in asymmetry due to differences between male and female within-sex correlations; the second term, $r_{\delta b}$, is due to asymmetry of the off-diagonal between-sex, between-trait correlations within the **B** matrix. Changing the direction of the concordant vector reverses the sign of the effects of the differences.

In general, traits will experience a mixture of antagonistic and concordant selection simultaneously. The overall rate of change in sexual dimorphism is a function of all the components of the **G** matrix. As the number of traits increases, the conditions under which the effects of concordant selection on dimorphism can exceed that of antagonistic selection of equal strength become more varied, as the asymmetries of the off-diagonal elements of $G_{M},\;G_{F}$, and **B**, as well as unequal genetic variances, can contribute to the indirect responses that affect dimorphism.

## Results

### Gene-Wise Analysis

Of the 12,071 genes investigated, we detected significant genetic variation in 10,489 genes, similar to the results obtained by Ayroles et al. (2009). We do not consider the nonsignificant genes further. Dimorphism of expression of the *i*th gene is quantified as $D_{i}$, the difference between means of log_2_-transformed male and female gene expression. Of the significant genes, 1,447 were male biased (MB: $D_{i}>1$, a ratio of male to female expression greater than 2), 2,073 were female biased (FB: $D_{i}<-1$, a ratio less than 0.5), and the remaining 6,979 genes had relatively unbiased expression (UB: ${\left|D\right|}_{i}\leq 1$, within a factor of two between the sexes).

### Which Aspects of G Correlate with Dimorphism of Transcription?

Under the familiar hypothesis that SAS drives the evolution of dimorphism, equations (3) and (6) predict that the absolute value of sexual dimorphism, |D|, will be positively related to $\bar{g}$ and negatively related to $r_{MF}$, as long as the **G** matrix is consistent over a relevant evolutionary time scale. In addition, ${\bar{r}}_{w}\;\text{and}\;{\bar{r}}_{b}$ have conflicting effects on the change in dimorphism. Their net effect can be positive or negative. If sexually concordant selection (SCS) influences dimorphism, equation (4) shows that differences in male and female genetic variances should be positively correlated with |D|, whereas equation (7) predicts that the absolute value of the average differences of within- $\left(\left|{\bar{r}}_{\delta w}\right|\right)$ and between-sex correlations $\left(\left|{\bar{r}}_{\delta b}\right|\right)$ should also be positively correlated with |D|. In addition, several authors (Mank et al. 2008; Assis et al. 2012; Griffin et al. 2013; Dean and Mank 2016) have shown that the mean level of gene expression, $\bar{E}$, and the degree of tissue-specificity, *τ* (Yanai et al. 2005), are related to |D|.

**Fig. 1.**
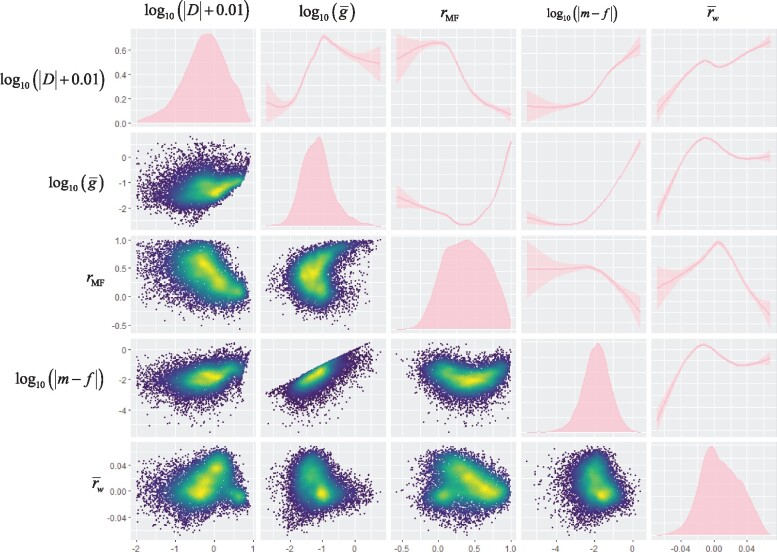
Exploratory distribution, density, and smoothed trend plots for $\;{\text{log}}_{10}\left(\left|\Delta \right|+0.01\right)$ and potentially predictive aspects of the **G** matrix. We chose the ggpairs function from R (R Core Team 2020) package GGally (Wickham 2016; Schloerke et al. 2020) to visualize the distribution and correlations among variables. These functions use the grammar of graphics philosophy (Wilkinson 2005) and emphasize visualization over quantification. Plots on the diagonal are density plots in which the horizontal axis is on the scale shown at the bottom of the plot, whereas the vertical axis is scaled to the maximum density. All other plots use the axes shown to the left as the vertical axis label, and below the figure as the horizontal axis label. Density estimates in the panels on the diagonal are kernel density estimates calculated in the geom_density function in ggplot2. Plots above the diagonal are functions ±95% CIs calculated in ggplot2, which calls a generalized additive model in the R package mgcv. Plots below the diagonal were visualized via 2D kernel estimates calculated by function geom_point_2d in the R package MASS (Venables and Ripley 2002). Color scale is based on quantiles of local density, and consequently, the scale varies from panel to panel. All calculations performed using function default parameters.

The Pearson correlation matrix of $\;{\text{log}}_{10}\left(\left|D\right|+0.01\right)$ and these predictors, calculated from the sample covariance matrix of sex-specific line means for all 10,489 genes, G*, is shown in supplementary table S1, Supplementary Material online. Note that statistical tests of association are invalid for these data, as the underlying expression data are not independently estimated for each gene. These relationships are nonlinear, and in some cases nonmonotonic, as shown in figure 1 for the relationship between dimorphism and a subset of these predictor variables. The predicted relationships of $r_{MF}\;\text{and}\;\;{\text{log}}_{10}\left(\bar{g}\right)$ with dimorphism under SAS are evident. In addition, ${\bar{r}}_{w}$ has a strong positive relationship with dimorphism, suggesting the effects of the within-sex between-trait correlations swamp those of the between-sex between-trait correlations captured by ${\bar{r}}_{b}$ (see eq. 6). Both $\;{\text{log}}_{10}\left(\left|m-f\right|\right)$ and $\left|{\bar{r}}_{\delta w}\right|$ have clear positive relationships with dimorphism as predicted under SCS (eqs. 4 and 7).

These raw relationships are confounded, as suggested by the complex relationships among predictor variables evident in supplementary table S1, Supplementary Material online, and figure 1. To help disentangle these factors, we used multiple regression of sexual dimorphism, measured as $\;{\text{log}}_{10}\left(\left|D\right|+0.01\right)$, on the other variables, bootstrapped at the level of inbred lines. The results are shown in table 1. The model explains 40% of the variation in dimorphism. Both mean expression $\bar{E}$ and tissue specificity *τ* have consistent positive effects on dimorphism, as shown by the positive signs of the bootstrap quantiles. $\;{\text{Log}}_{10}\left(\bar{g}\right),\;r_{MF}\;\text{and}\;{\bar{r}}_{w}$ explain 2.1%, 15.5%, and 6.7% of the variance in $\;{\text{log}}_{10}\left(\left|D\right|+0.01\right)$, respectively. In addition, there is also a consistent positive relationship of $\;{\text{log}}_{10}\left(\left|m-f\right|\right)$ with $\;{\text{log}}_{10}\left(\left|D\right|+0.01\right)$, as expected under SCS, explaining 1.1% of the variance. Expression properties $\bar{E}$ and *τ* also have significant positive relationships with $\;{\text{log}}_{10}\left(\left|D\right|+0.01\right)$, explaining 3.2% and 3.5% of the variation.

**Table 1.** Estimates of Slopes from Multiple Regression of $\;{\text{log}}_{10}\left(\left|D\right|+0.01\right)$ on Expression Characteristics, Bootstrapped over Genes.

		All Genes			Biased^d^			Unbiased^d^		
Parameter^a^	Pred.^b^	Median	Quantiles^c^ 2.5%, 97.5%	*R* ^2^	Median	Quantiles^c^ 2.5%, 97.5%	*R* ^2^	Median	Quantiles^c^ 2.5%, 97.5%	*R* ^2^
$\bar{E}$		**0.04**	**0.03, 0.05**	**3.2**	**0.03**	**0.02, 0.03**	**6.2**	**0.03**	**0.03, 0.04**	**1.8**
*τ*		**0.78**	**0.49, 0.96**	**3.5**	**0.99**	**0.82, 1.12**	**32.6**	−0.01	−0.10, 0.10	0.0
$\;{\text{log}}_{10}\left(\bar{g}\right)$	A+	**0.20**	**0.02, 0.34**	**2.1**	0.05	−0.04, 0.11	0.8	**0.14**	**0.10, 0.19**	**1.2**
${\bar{r}}_{MF}$	A−	**−0.63**	**−0.76, −0.48**	**15.5**	**−0.10**	**−0.15, −0.04**	**4.5**	**−0.28**	**−0.38, −0.19**	**2.3**
$\;{\text{log}}_{10}\left(\left|m-f\right|\right)$	C+	**0.11**	**0.07, 0.16**	1.1	0.00	−0.02, 0.02	0.3	**0.04**	**0.02, 0.07**	**0.4**
${\bar{r}}_{w}$	A?	**4.81**	**2.99, 7.66**	**6.7**	**0.41**	**0.19, 0.60**	**0.3**	−0.95	−2.13, 0.50	4.2
${\bar{r}}_{b}$	A?	0.03	−1.52, 1.41	0.1	0.09	−0.08, 0.30	0.2	0.15	−0.49, 0.96	0.0
$\left|{\bar{r}}_{\delta w}\right|$	C+	3.72	−6.10, 14.23	1.8	0.01	−0.44, 0.52	1.3	1.17	−1.58, 5.59	0.6
$\;{\text{log}}_{10}\left(\left|{\bar{r}}_{\delta b}\right|+0.01\right)$	C+	−0.09	−0.23, 0.20	0.0	−0.00	−0.08, 0.13	0.4	−0.07	−0.14, 0.01	0.1

Note.—A, antagonistic prediction; C, concordant prediction;?, both ${\bar{r}}_{w}$ and ${\bar{r}}_{b}$ are predicted to affect dimorphism under SAS, but the sign of these effects depends on the details of selection.

a Parameter symbols explained in the text; ${\bar{r}}_{w}\;\text{and}\;{\bar{r}}_{b\;}$ generalize the indirect selection parameters defined in equation (6) to include the mean of the average within-sex correlations of all traits with expression of the focal gene, whereas $\left|{\bar{r}}_{\delta w}\right|\;\text{and}\;\left|{\bar{r}}_{\delta b}\right|$ generalize those in equation (7) to include the absolute values of the mean differences of all within-sex correlations with the focal gene, or the differences in the between-sex correlations involving the focal gene. $\left|{\bar{r}}_{\delta b}\right|$ was log-transformed to minimize the influence of observations with exceptionally high values.

b Predicted sign of relationships with $\;{\text{log}}_{10}\left(\left|D\right|+0.01\right)$ under concordant or antagonistic selection.

c Quantiles from 1,000 bootstrap resamples at the inbred line level. When the bootstrap 95% quantiles have consistent sign, we consider the effects to be statistically significant. Significant values are shown in bold face.

d Biased genes have |D|>1; Unbiased genes |D|≤1.

Separate analyses of the biased (|D|>1) genes show that the **G** matrix properties are much less predictive of dimorphism in this subset. $\bar{E}$ and *τ* remain significant, and the role of *τ* is much stronger in this class of genes, explaining 32.6% of the variation. Of the **G** matrix properties, $r_{MF}$ and ${\bar{r}}_{w}$ remain consistent predictors of dimorphism, although the variance explained is much reduced from that over the entire data set. Analysis of just the relatively unbiased (|D|≤1)genes shows that $\bar{E}$ is again a significant predictor, but *τ* is not. **G** matrix properties expected to covary with $\;{\text{log}}_{10}\left(\left|D\right|+0.01\right)$ under both SAS and SCS are significant in this subset; the relationships with $\;{\text{log}}_{10}\left(\bar{g}\right)\;\text{and}\;r_{MF}$ suggest the action of SAS, whereas the positive relationship with $\;{\text{log}}_{10}\left(\left|m-f\right|\right)$ is consistent with SCS. All of these variables explained a small proportion of the variance in dimorphism. On balance, these results indicate a strong signature of past SAS over the entire range of dimorphism values, and a more modest effect of SCS which is strongest for traits that have smaller amounts of dimorphism.

Innocenti and Morrow (2010) estimated selection on expression for a subset of the transcripts in our data set, but unfortunately did not report selection effect sizes. Transcripts with a significant sex by fitness interactions (their models used transcription as the dependent variable) experience some SAS, whereas those with a significant main effect of fitness experience some SCS. Note that selection may include both concordant and antagonistic components (Cheng and Houle 2020). When the identity of genes with a fitness main effect, or a sex-by-fitness interaction, were entered as predictors in the multiple regression model, these terms had small negative effects on |D| (median, 2.5 and 97.5 percentiles: main effect *b*= −0.034, −0.056, −0.011; interaction *b*= −0.057, −0.073, −0.034). Perhaps more importantly, the parameter estimates shown in table 1 were essentially unchanged by the fitness effect indicators. There is no clear predicted relationship between the selection measured in the lab by Innocenti and Morrow (2010) and dimorphism. One potential explanation for the negative relationship between selection and dimorphism is that Innocenti and Morrow’s study had greater power to detect selection in less dimorphic transcripts.

### Quantitative Genetic Analysis of Gene Expression

Ayroles et al. (2009) measured gene expression in just 40 inbred lines, which precludes estimating a **G** matrix for more than a small number of traits. To choose these traits, we performed principal component analyses within the male-, female-, and relatively-unbiased gene classes, as described in the Methods. Through exploratory analyses described in supplementary results, Supplementary Material online, we chose the first two PCs of male-biased genes (symbolized MB), and of female-biased genes (FB), and the first four PCs for relatively unbiased genes (UB). These analyses revealed no evidence for genetic variance in female expression of MB genes, or for male expression of FB genes, suggesting that direct selection to alter expression in the low-expressing sex will be relatively ineffective. Accordingly, we dropped these traits from **G**. The transcriptional modules inferred by Ayroles et al. (2009) are not generally associated with the sex-bias class PCs used in the quantitative genetic analysis (see supplementary methods, results, and table S4, Supplementary Material online).

The best-fitting model suggested that genetic variation in the 12 traits can be explained well by a 9 dimensional model, resulting in the **G** matrix shown in table 2. The resulting data set consisted of 12 traits, although only the four UB (UB1–UB4) traits were estimated in both sexes. Thus, only the portions of table 2 involving these traits (outlined in boxes) correspond to Lande’s (1980)**B** matrix. For these UB traits, $r_{MF}$ averages 0.82. This is higher than the average value of 0.75 found in a review of previous studies (Poissant et al. 2010), and much higher than the average *r*_MF_ value of estimated in Griffin et al. (2013). Three of these correlations are less than 3 SE from a perfect correlation of 1, suggesting that there may be very little variation to generate opposing responses in the sexes for these traits (Sztepanacz and Blows 2017).

**Table 2.** Genetic Correlation and Covariance Matrices from the 12 Trait Analyses.


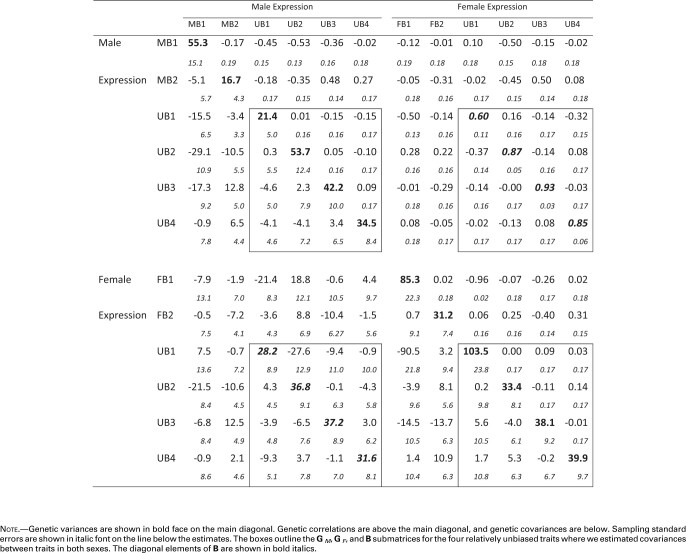

**Figure:**
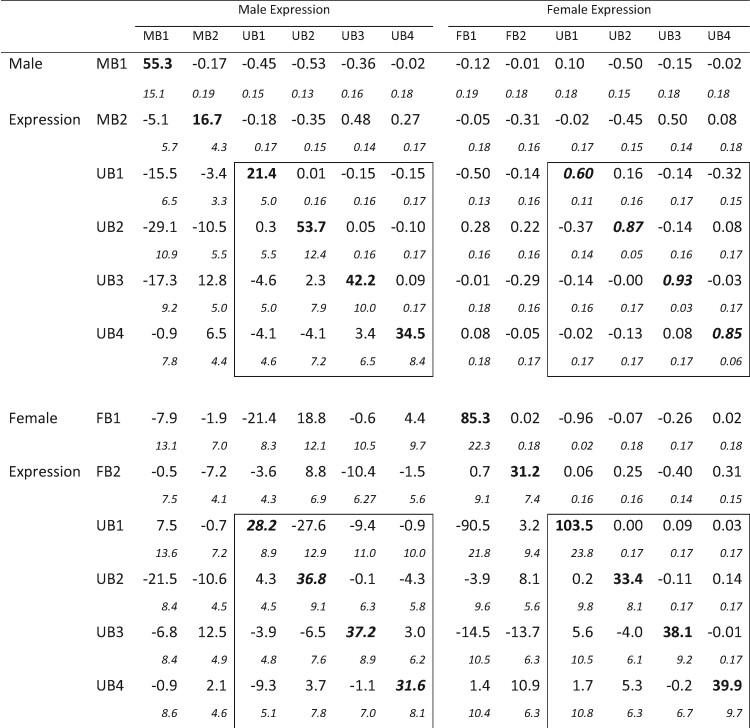
**Table 2**. Genetic Correlation and Covariance Matrices from the 12 Trait Analyses. Note.—Genetic variances are shown in bold face on the main diagonal. Genetic correlations are above the main diagonal, and genetic covariances are below. Sampling standard errors are shown in italic font on the line below the estimates. The boxes outline the **G**_*M*_, **G**_*F*_, and **B** submatrices for the four relatively unbiased traits where we estimated covariances between traits in both sexes. The diagonal elements of **B** are shown in bold italics.

Examination of table 2 also reveals several other correlations indicative of constraints on the evolution of biased gene expression. The most striking of these is female expression of FB1 and of UB1 where r=−0.96. In addition, many of the correlations between MB traits and male expression of the relatively unbiased traits have absolute values in the neighborhood of 0.4–0.5, as does the correlation of FB2 with female expression of UB3. These elements show the potential for complex constraints between unbiased and biased genes.

### Predicted Responses to Selection

We used **G** to predict the effects of current selection on changes in dimorphism from its current level. We predicted the responses to symmetrical antagonistic and concordant versions of five different selection gradients: directional selection on each of the four UB traits individually, and directional selection on all four simultaneously with the results shown in table 3. We show two different estimates of the amount of evolution under each selective regime. Evolvability, *e*, is the response in the direction of the selection gradient, whereas respondability, *R*, is the length of the total response to selection (Hansen and Houle 2008). As expected from the positive *r*_MF_, the responses to SCS selection are overall larger than the responses to SAS.

**Table 3.** Responses of Relatively Unbiased Expression Traits to Antagonistic (A) and Concordant (C) Selection of Equal Strength (‖β‖=1).

	Antagonistic^b^		Concordant^c^		UB ‖Δ‖^d^			Vector correlation. $\Delta {\bar{z}}_{M}\;\text{vs}.\;\Delta {\bar{z}}_{F}$	
Sel.^a^	*E*	*R*	*e*	*R*	A	C	ratio	A	C
UB1	35.6 (21.0–52.9)	60.6 (35.6–89.2)	90.8 (53.1–133.7)	104.7 (62.9–155.0)	27.4 (16.3–40.5)	31.2 (16.0–49.4)	0.87 (0.66–1.26)	0.27 (−0.22 to 0.60)	0.83 (0.65–0.97)
UB2	7.9 (4.1–12.7)	24.9 (14.0–38.2)	87.7 (55.1–132.0)	94.1 (60.0–140.6)	10.4 (5.7–16.1)	16.1 (6.6–29.7)	0.63 (0.34–1.46)	0.34 (−0.09 to 0.60)	0.89 (0.73–0.98)
UB3	4.2 (1.9–7.5)	15.2 (6.8–24.9)	84.4 (50.6–125.7)	88.6 (54.0–130.1	6.3 (2.8–10.6)	7.5 (3.0–16.5)	0.81 (0.29–2.24)	0.33 (−0.30 to 0.73)	0.97 (0.88–1.00)
UB4	7.0 (3.7–11.5)	16.2 (8.9–25.8)	73.0 (45.2–108.8)	77.4 (48.5–114.3)	6.2 (3.2–10.7)	7.2 (2.3–18.7)	0.86 (0.28–2.85)	0.39 (−0.52 to 0.80)	0.97 (0.82–1.00)
UB	27.6 (17.3–40.4)	48.9 (28.4–73.3)	70.4 (44.7–103.5)	75.9 (48.7–110.6)	23.8 (14.7–34.7)	13.2 (3.8–26.2)	1.80 (0.93–5.42)	0.10 (−0.46 to 0.46)	0.92 (0.80–0.99)

Note.—Values are medians (2.5–97.5% quantiles). UB, all four UB traits are simultaneously selected; *e*, evolvability, the response in the direction of selection; *R*, respondability, the total response to selection; A, C, total change in dimorphism under A or C selection; ratio, $\left\|\Delta_{A}\right\|/\left\|\Delta_{C}\right\|$.

a Selection regime: symbols indicate trait subject to directional selection.

b Selected male traits have positive gradients, whereas female traits negative selection gradients.

c All selected traits have positive gradients in both sexes.

d Predicted change in length of dimorphism vector.

The key prediction concerns the total change in sexual dimorphism, the change in the length of the multivariate vector of differences between the sexes, ‖Δ‖. Surprisingly, ‖Δ‖ is often larger under SCS than under SAS. Although the confidence limits of the ratios of ‖Δ‖ due to SAS and SCS are not significantly different from one for any of the selection gradients investigated, these results contrast with the generally assumed scenario that sexual dimorphism is the result of direct selection for dimorphism. As shown in the last two columns of table 3, responses of each sex to SAS are positively correlated, rather than negatively correlated, as selection favors.

To get a sense for how much dimorphism can be created by concordant selection, we can compare the direct response to concordant selection with the indirect response in dimorphism in table 3. These ratios range from 0.34 to 0.09 for the four UB traits. The total change in dimorphism when trait UB1 is concordantly selected, for example, is more than 1/3 as much (‖Δ‖=31.2) as the total concordant change (*e *=* *90.8) in trait UB1. Thus, large changes in average gene expression can create smaller but still substantial total changes in dimorphism.

We also predicted the response of dimorphism of the relatively unbiased traits to selection on the sex-biased MB and FB traits with results shown in supplementary table S5, Supplementary Material online. The total changes in dimorphism under these scenarios are of comparable magnitudes to the changes arising from selection on the UB traits. Indirect responses of dimorphism to selection on sex-biased traits can have important effects on other traits.

The scenarios in table 3 and supplementary table S5, Supplementary Material online, assume that there is no selection on the traits not under directional selection. Alternatively, we can assume that traits not under directional selection are subject to such strong stabilizing selection that a response is only possible in the direction of the selection gradient (Hansen et al. 2003; Hansen and Houle 2008). Under this sort of selection, SAS and SCS again have similar effects on overall dimorphism as shown in supplementary table S6, Supplementary Material online, although the responses are much less than for the corresponding unconditional scenarios in table 3.

The results in table 4 show how these predictions are affected by the symmetry of **G.** To make these comparisons, we substituted the symmetrical version of $G_{M}\;\text{and}\;G_{F}$ (their average, $\bar{G}$) and **B **(**B_*S*_**) into the **G** matrix and compared the predicted response to those from the unaltered matrix. Substituting $\bar{G}$ reduces the change in dimorphism in response to SCS by an average of 30.4% over our five selective scenarios. Symmetrizing **B** can either increase or decrease the dimorphic response to SCS, but the average changes in dimorphic response are reduced by 28.9%, nearly as large as the effect of symmetrizing **G**_*M*_ and **G**_*F*_. Interestingly, the asymmetries due to **B, G**_*M*_, and **G**_*F*_ can have opposite effects. For example, simultaneous SCS on all UB traits leads to smaller changes in dimorphism relative to that under SAS than any other selection regime. The results in table 4 show that symmetrizing **B** increases dimorphism under SCS by 41%, while symmetrizing **G**_*M*_ and **G**_*F*_ decreases dimorphism by 41%. The change in dimorphism is small because the asymmetries work against each other. The predicted response in dimorphism in UB2, however, is large because both types of asymmetry affect dimorphism in the same direction. In addition, the effects of these asymmetries are highly nonlinear in combination, as eliminating both kinds of asymmetry eliminates the evolution of dimorphism under concordant selection, regardless of how each asymmetry separately affects dimorphism. As predicted, symmetrizing the matrices has no effect on the change in dimorphism under SAS. The **B** matrix as a whole is an important constraint to the evolution of dimorphism, as eliminating it always increases the change in dimorphism under SAS.

**Table 4.** Ratio of Changes in Relatively Unbiased Transcript Dimorphism (‖Δ‖) Predicted from a Modified **G** Matrix Relative to Predictions from the Unmodified **G** Matrix.

	(UB ‖Δ‖ Modified G)/(UB ‖Δ‖ Unmodified G)^b^									
	$\left[\begin{array}{cc} \bar{G} & B\\ B^{T} & \bar{G} \end{array}\right]$		$\left[\begin{array}{cc} G_{M} & B_{S}\\ B_{S} & G_{F} \end{array}\right]$		$\left[\begin{array}{cc} G_{M} & 0\\ 0 & G_{F} \end{array}\right]$		$\left[\begin{array}{cc} \bar{G} & B_{S}\\ B_{S} & \bar{G} \end{array}\right]$		$\left[\begin{array}{cc} \bar{G} & 0\\ 0 & \bar{G} \end{array}\right]$	
Sel.^a^	A	C	A	C	A	C	A	C	A	C
UB1	1.00	0.37	1.00	0.93	1.81	0.91	1.00	0.00	1.68	0.00
UB2	1.00	0.77	1.00	0.53	2.53	0.27	1.00	0.00	3.13	0.00
UB3	1.00	0.94	1.00	1.32	4.67	1.04	1.00	0.00	4.94	0.00
UB4	1.00	0.81	1.00	0.84	5.21	0.91	1.00	0.00	5.36	0.00
UB	1.00	0.59	1.00	1.41	1.33	1.38	1.00	0.00	1.48	0.00

a Selection regime (as in table 3).

b See text for explanation of modified **G** matrices.

## Discussion

Most previous analyses of the relationship between sexual dimorphism and genetic variation have made two limiting assumptions (e.g., Poissant et al. 2010; Griffin et al. 2013). The first is that sexual dimorphism is shaped by direct, sexually antagonistic selection (SAS) that favors dimorphism. The second is that genetic constraints on the evolution of dimorphisms can be well–characterized by a single parameter, the intersexual genetic correlation of each trait, $r_{MF}.$ The latter assumption is as much practical as conceptual: there are relatively few estimates of sex-specific multivariate **G** matrices, compared with the bivariate ones.

We reanalyzed genetic variation in gene expression in *D. melanogaster* (Ayroles et al. 2009) to include the effects of other types of selection, and to capture the effects of other aspects of inheritance on the evolution of dimorphism. When we predict the ability of gene expression to respond to contemporary selection, sexually concordant selection (SCS) that selects the phenotypes of each sex in the same direction is equally capable of causing the evolution of sexual dimorphism as SAS that selects the sexes in opposite directions. When we use the current **G** matrix to retrodict the current level of dimorphism, we confirm that $r_{MF}$ is strongly and negatively correlated with dimorphism, as expected under SAS. However, we also find that aspects of the **G** matrix predicted to result in changes in dimorphism under SCS are also correlated positively with current dimorphism. In addition, we highlight aspects of genetic variation, other than the values of $r_{MF},$ that affect the evolution of dimorphism of gene expression.

Asymmetry in genetic variances between the sexes has long been predicted to cause dimorphism in response to SCS (Fisher 1930; Lande 1980; Leutenegger and Cheverud 1982; Cheverud et al. 1985; Lynch and Walsh 1998, chapter 24; Bonduriansky and Chenoweth 2009; Wyman et al. 2013), but the role of such differences has rarely been considered in relation to observed sexual dimorphisms. An exception is Leutenneger and Cheverud’s (1982) proposal that dimorphism of primate canine teeth and body weight is caused by indirect response to selection on an average body size mediated by differences in genetic variances between the sexes.

Our analysis of the two trait case, as well as a more comprehensive analysis of the *k* trait case (Cheng and Houle 2020), shows that asymmetries in the cross-trait covariances between the male and female genetic variance–covariance (**G**) matrices, and asymmetries within the cross-sex covariance (**B**) matrix also play a role in the evolution of dimorphism. The one-trait analyses that feature $r_{MF}$ omit the role of these aspects of the **G** matrix by assumption. In general, these cross-trait covariances can play a large role in shaping evolutionary trajectories (Blows and Hoffmann 2005; Hansen and Houle 2008; Walsh and Blows 2009). Their omission from existing analyses of gene expression data is unfortunate, as these data give us the opportunity to address effects of indirect selection at an unprecedented scale. Critically, the asymmetries of cross-trait covariances within **G** matrices promote the evolution of dimorphism under concordant selection, and are irrelevant under antagonistic selection. In contrast, the response of dimorphism to antagonistic selection depends only on the average of the male and female **G** matrices, and the symmetrical part of the **B** matrix.

To investigate the effects of past selection on current sexual dimorphism, we estimated the **G** matrix for all of the genes with significant genetic variation in gene expression, which we term G*. As in previous studies (e.g., Griffin et al. 2013), there was strong evidence that between sex-correlations were negatively related to dimorphism. In addition, the average within-sex genetic variance of the focal trait and average within-sex correlations of the focal trait with all other traits were positively related to dimorphism. Our novel finding is that aspects of G* that predict changes in dimorphism under concordant selection, such as the difference between male and female trait variances, are also correlated with current levels of dimorphism, although to a lesser extent than those that predict dimorphism under antagonistic selection. Rigorous statistical testing of these results is not possible as the expression of individual genes is not independent.

To predict the possible effects of current selection on sexual dimorphism, and overcome the lack of independence of expression among genes, we generated unbiased estimates of **G** for a relatively small number of linear combinations of expression traits that captured the major axes of variation in female-biased, male-biased, and relatively unbiased genes. For five of these eight traits, genetic variation in males and females was highly asymmetrical. This alone will cause the evolution of dimorphism under any selection regime with a concordant component. Predictions of response to selection based on the unbiased **G** matrix showed that SCS and SAS are approximately equally capable of changing sexual dimorphism. The same result holds for selection that includes nonlinear components that restrict the evolution of some traits, while favoring changes in others. If antagonistic and concordant selection are equally strong, they will contribute roughly equally to dimorphism in the responses of gene expression to selection. In addition, the indirect responses due to cross-trait correlations were sometimes quite substantial. For example, selection on the highly dimorphic genes is predicted to change the dimorphism in the less–dimorphic genes by a comparable amount to direct selection on the less–dimorphic genes.

A substantial body of evidence suggests differences in the evolution of expression in male- versus female-biased genes (Allen et al. 2018). Interspecific evolution of male-biased gene expression is more rapid (Ellegren and Parsch 2007), male-biased genes have more genetic variance, greater tissue-specificity and higher intersexual correlations than female-biased genes (Mank et al. 2008; Assis et al. 2012; Allen et al. 2018). In contrast, our quantitative genetic analyses show that the genetic variance of female-biased traits in females is higher than the genetic variance of male-biased traits in males. We were unable to accurately estimate the intersexual correlations in sex-biased genes, as we found no significant genetic variation in the less-highly expressing sex for these genes. However, the single largest genetic correlation in our matrix was between a female-biased expression trait and female expression of an unbiased expression trait. A better-estimated **G** matrix will be necessary to address the evolutionary differences between the male- and female-biased genes.

Summarizing these empirical results, analysis of aggregate measures of genetic variation suggests that substantial variation in dimorphism can be created by directional selection, regardless of whether that selection is antagonistic or concordant. In addition, the dimorphism of individual genes shows traces of both antagonistic and concordant selection.

Lande’s (1980) original explication of the quantitative genetics of dimorphism clearly incorporated the likelihood that concordant selection would affect dimorphism. Lande chose to emphasize the effects of antagonistic selection for dimorphism based on the assumption that evolution of sex-averaged means would be relatively unconstrained and rapidly achieve their optima, whereas evolution of differences between the sexes would tend to be constrained, and take a long time to evolve to their optima. This point is made explicit in the model of Connallon and Clark (2014) who show that the evolution of some dimorphism is almost inevitable whenever selection perturbs any initially monomorphic population. In this case, even if changes in trait optima are random, sex-specific traits will tend to be under antagonistic selection more frequently than they are under concordant selection.

A key motivation for our work is the observation that SAS is rarer than SCS, and, more importantly, is relatively weak when it is observed (Cox and Calsbeek 2009; Morrissey 2016). The available data on which to base this conclusion are admittedly rather weak (Cheng and Houle 2020), but unless this conclusion is rejected by future studies, it is clear that we must take seriously the possibility that some sexual dimorphism is just the byproduct of selection for other trait changes.

Lande’s (1980) argument that concordant selection should be rare and that antagonistic selection persistent is based on two complementary arguments. One is that selection regimes change infrequently, allowing the population to approach sex-averaged optima. The alternative is that changes in the direction of concordant selection often take place before a sex-averaged optimum can be achieved. This alternative scenario is consistent with the pattern of sex-specific selection identified by Morrissey (2016), as well as the frequent observation of very strong linear selection gradients in many populations (Kingsolver et al. 2001; Hereford et al. 2004). A second assumption is that sexual dimorphism is under strong selection, at least when perturbed away from the optimum state. When sexual dimorphism is weakly selected, the dimorphism created by indirect responses to selection may persist. One simple scenario for weak selection on dimorphism is selection for a minimum level of gene expression in one sex, with very small fitness costs to additional expression, whereas selection favors higher expression in the other sex. In this case, directional selection on just one sex creates equal selection for concordant and antagonistic changes (Cheng and Houle 2020).

In conclusion, several aspects of our results suggest the possibility that sexual dimorphism of gene expression in *D. melanogaster* may reflect the indirect effects of concordant selection on the dimorphic traits, or the indirect effects of selection on other traits. Our estimate of the **G** matrix suggests many unexpected correlations and asymmetries that will together generate dimorphism under any selective regime, as well as under genetic drift. Strong selection on one aspect of gene expression will frequently generate widespread perturbations in the expression of genes that are not directly selected. The upshot of these factors is that dimorphism in the expression of any particular gene cannot be assumed to be adaptive. We do not doubt that many aspects of transcription do reflect persistent selection for sexual dimorphism. Deciding which aspects of dimorphism are so selected requires more detailed analyses than have so far been applied.

## Materials and Methods

### Gene Expression Data

We reanalyzed the adult gene expression (Ayroles et al. 2009) in inbred lines of the Drosophila Genome Reference Project (Mackay et al. 2012). Each DGRP line was independently derived from a different inseminated female *D. melanogaster* sampled from a single outbred population, followed by 20 generations of brother–sister mating.

Ayroles et al. (2009) assayed whole-body gene expression using Affymetrix Drosophila Genome 2.0 microarrays. They assayed expression twice in each sex in each of 40 DGRP lines, for a total of 160 chips. The data were normalized and processed using the R Bioconductor package *oligo* (Carvalho and Irizarry 2010; Huber et al. 2015). In some cases, a single probe assayed expression of transcripts at more than one gene. We assigned results from such probes to just one of these genes, chosen arbitrarily. This left expression data on 12,701 genes. Gene expression was in log_2_ units, so differences in expression are equivalent to log_2_(ratios).

### Gene-by-Gene Analyses

We tested for the presence of significant genetic variation at each gene using a mixed model analysis with sex, probe, and probe-by-sex effects fixed, and line, line-by-sex and line-by-probe effects treated as random. For 11,039 genes, a single probe was assayed, and the probe effects were omitted from the model. We compared the likelihood of the data for models that included or omitted all the random effect terms using a likelihood ratio test with 2 df for the genes with only one probe, and three df for genes with more than one probe. Genes were retained for further analysis if *P < *0.01 for the total line effects. Mixed model analyses were fit using Proc Mixed in SAS/STAT software (SAS Institute Inc 2016).

For genes with significant line effects, we retained the least squares means from this model for each sex and line, and then calculated the covariance matrix of these means to form G*. Sexual dimorphism in expression of the *i*th gene, $D_{i}={\bar{z}}_{M\cdot i}-{\bar{z}}_{F\cdot i}$, was measured as the difference between the least squares means of male and female log_2_-transformed gene expression. We defined three categories of sex bias: male-biased (MB) genes with male expression more than twice as large as female expression, $D_{i}>1$, female-biased (FB) genes with female expression more than twice as large as male expression, $D_{i}<-1$, and relatively unbiased (UB) genes with $\left|D_{i}\right|\leq 1$.

From equation (3), we predict that if antagonistic selection plays a major role, $\left|D_{i}\right|$ will be positively related to ${\bar{g}}_{i}$, and negatively related to $r_{\text{MF.}i}$. From equation (4), we predict a positive relationship between $\left|m_{i}-f_{i}\right|$ and $\left|D_{i}\right|$ under concordant selection. From equation (6), we can see that the averages of the corresponding off-diagonal elements of $G_{M}^{*}\;\text{and}\;G_{F}^{*}$, and of off-diagonal elements above and below the diagonal of B* affect the evolution of dimorphism under antagonistic selection. To capture the effect of within-sex correlations for the *i*th gene, we calculated:

$${\bar{r}}_{w.i}=\left(\sum_{i\neq j}r_{MiMj}+r_{FiFj}\right)/\left(2\left(n-1\right)\right),$$

where the summation is over all genes, and *n* is the number of genes. To summarize the effects of between-sex correlations, we used:

$${\bar{r}}_{b.i}=\left(\sum_{i\neq j}r_{MiFj}+r_{FiMj}\right)/\left(2\left(n-1\right)\right)$$

From equation (7), we can see that differences of the corresponding off-diagonal elements of $G_{\text{M}}^{*}\;\text{and}\;G_{\text{F}}^{*}$, and of off-diagonal elements above and below the diagonal of B* promote the evolution of dimorphism under concordant selection. We quantified the effects of the differences in within-sex correlations as:

$$\left|{\bar{r}}_{\delta w.i}\right|=\left|\left(\sum_{i\neq j}r_{MiMj}-r_{FiFj}\right)/\left(2\left(n-1\right)\right)\right|,$$

and the differences in between-sex correlations as:

$$\left|{\bar{r}}_{\delta b.i}\right|=\left|\left(\sum_{i\neq j}r_{MiFj}-r_{FiMj}\right)/\left(2\left(n-1\right)\right)\right|.$$

In addition, we also investigated the effects of the mean level of expression, ${\bar{E}}_{i}$, and an index of the tissue specificity of gene expression, *τ_i_* (Yanai et al. 2005), on the level of sexual dimorphism. We downloaded tissue-specific expression data from http://flyatlas.org/ Geo accession GSE7763 (last accessed January 5, 2021), and calculated *τ* for each transcript, then averaged them to obtain *τ_i_* for gene *i*.

Values of $\left|D_{i}\right|$ must be positive and are strongly right-skewed with a small minority of very large values. To reduce the influence of this large tail, while preventing values very near 0 from causing a left-skew to the transformed data, we transformed dimorphism as $\;{\text{log}}_{10}\left(\left|D_{i}\right|+0.01\right)$. We log_10_-transformed ${\bar{g}}_{i}$, $\left|m_{i}-f_{i}\right|$ and $\left|{\bar{r}}_{\delta b.i}\right|$ before analysis for similar reasons.

### Selection on Gene Expression in *Drosophila melanogaster*

The nature of selection on transcripts was derived from table S1 in Innocenti and Morrow (2010). For 15 genotypes with high and low male and female fitnesses, they tested whether fitness predicted the expression of each transcript, and for a sex by fitness interaction. They only report *P* values for tests judged to be significant, so it is unclear how many total transcripts were tested. When paired with the Ayroles et al. (2009) data set, 516 genes had least one transcript with a significant main effect, 1,300 had a significant interaction, and 348 had both effects significant.

### Quantitative Genetic Analysis of Bias Classes

We performed separate principal component analyses (PCA) on the covariances of least-squares means of expression in each sex for the male-, female, and relatively-biased genes. For the unbiased genes, we performed a PCA on the covariance matrix of line-sex averages. For the two biased sets, we performed a PCA of expression in the dominant sex. We partitioned the variance in the principal component scores into genetic and nongenetic sources using restricted maximum-likelihood implemented in the program Wombat (Meyer 2006–2019). We assumed that all the inbred lines were unrelated. Whole-genome sequencing of these lines suggests that this is a good, albeit imperfect, approximation of the relationship among them (Huang et al. 2014). Estimation of the genetic variance–covariance matrix, **G**, was carried out for both full- and reduced-rank models (Kirkpatrick and Meyer 2004; Meyer and Kirkpatrick 2005, 2008), and we selected the best-fitting model on the basis of Akaike’s information criterion corrected for small sample size (AICc).

We assessed the fit of models with different numbers of traits drawn from the three expression bias classes, eventually settling on a 12-trait data set as described in the Results section. Sampling variances of matrix elements remained stable under models fit to even fewer traits. Once we obtained well-estimated matrices, we back-transformed estimates to the original scores and used the REML-MVN approach (Meyer and Houle 2013; Houle and Meyer 2015) to generate 1,000 replicate matrices drawn from the sampling distribution of the matrix **G**. These replicate estimates of **G** were used to generate the sampling distribution of the predicted responses to selection. In most cases, the distributions of **G**-derived statistics were asymmetrical, so we report medians and 2.5% and 97.5% quantiles.

### Analyses of Modified G Matrices

To investigate which aspects of **G** matrix structure have effects on the evolution of dimorphism, we formed five modified matrices that highlight those aspects of **G** that affect the evolution of dimorphism. The *k *=* *2 theory developed above, as well as the more general analyses of Cheng and Houle (2020) suggests that the evolvability of dimorphism to antagonistic selection is promoted by the average genetic variance in males and females, $\bar{G}=(G_{M}+G_{F})/2$, and restricted by the symmetrical component of $B,\;B_{S}=\left(\text{B}+B^{T}\right)/2$. When these matrices are substituted for $G_{M},\;G_{F},\;B\;\text{and}\;B^{T}$ this eliminates the components of **G** that cause evolvability of dimorphism under concordant selection, namely the asymmetry between $G_{M}\;\text{and}\;G_{F},\;\left(G_{M}-G_{F}\right)/2$ and the asymmetrical component of $B,\;B_{A}=\left(B-B^{T}\right)/2$. In addition, we explored the effect of eliminating **B** by replacing it with the zero matrix **0**.

## Supplementary Material

Supplementary data are available at *Molecular Biology and Evolution* online.

## Supplementary Material

msaa329_Supplementary_Data
