# Supplementary material for: Predicting the Evolution of Sexual Dimorphism in Gene Expression
Source: Mol Biol Evol. 2021 Jan 29;38(5):1847–59. doi: 10.1093/molbev/msaa329 (PMC8097294; doi:10.1093/molbev/msaa329)
Supplement: msaa329_Supplementary_Data [file msaa329_supplementary_data.pdf]

Supplementary Material for

Predicting the evolution of sexual dimorphism in gene expression

David Houle[1*] and Changde Cheng[2†]

[1] Department of Biological Science, Florida State University, Tallahassee, Florida 32306, USA

[2]Department of Integrative Biology, University of Texas, Austin, Texas 78712, USA

*Corresponding author. E-mail dhoule@bio.fsu.edu

[†] Present address: Department of Computational Biology, St. Jude Children's Research Hospital, Memphis, Tennessee 38105, USA

This file contains Supplementary Methods and Results, Supplementary Tables S1-S6.

Supplementary Methods

Relationship between modules and bias-specific eigenvectors

Ayroles et al. (2009) identified 241 transcriptional modules in their data. To determine the relationship between these modules and the eigenvectors used to summarize the variation for the quantitative genetic analysis, we split the module data into the male- female- and relatively-unbiased classes. For each class, we formed a vector of indicator variables for each gene, with a one for each gene in the module and zeroes otherwise, then standardized the elements of this vector to have mean 0. We then calculated the vector correlation between this module-indicator vector and the absolute value of each eigenvector. Nominal significance of these correlations was evaluated relative to a null distribution of each indicator vector with 1000 random vectors with elements drawn from a normal distribution. We investigated correlations with modules that had 50 or more genes.

Supplementary Results

Choosing composite traits for quantitative genetic analysis

To choose informative traits, we extracted three submatrices from **G*:** male expression of male biased genes (MB), female expression of female biased genes (FB), and the sex-averaged expression of the remaining genes, termed relatively unbiased (UB). We then conducted a principal components analysis of submatrix. Male and female expression of each line was then scored on the first two PCs in the MB and FB subsets, and the first four in the UB subset, so k=8 in each sex.

Analyses of genetic variation of the k=8 data showed that the best fitting model had eight variable dimensions vectors out of 16 possible (and fit more than 3.6 AICc units better than the 7- or 9- dimensional models). The covariance and correlation matrices estimated using this model are shown in Supplementary Table S2. The average information matrix contained very small elements that prevented estimation of standard errors for all model terms, suggesting that the overall fit of the model was poor.

To help diagnose the trait combinations lacking significant genetic variation, we examined the estimates of trait variances, shown in Supplementary Table S3. Sex-specific variances are substantially different for the biased expression classes (MB and FB). Note that these biased traits as well as trait UB1 have very large asymmetries in the sex-specific genetic variances, making d (equation 3) substantially less than 1. This indicates that dimorphism is much more likely to evolve under both SAS (equation 3) and SCS (equation 4) than under the assumption of equal variances.

We conjectured that the female variances for the male-based traits and the male variances for the female-biased traits are not distinguishable from 0. To test this, we dropped the female expression of male-biased traits and the male expression of female-biased traits from the data set, and repeated estimation of the genetic variance for the remaining 12 variables. The best model now had nine significant genetic dimensions, although this was just 0.91 AICc units better than the eight-dimensional model. This supports the hypothesis that there is no significant genetic variation in females for male-biased genes or in males for female biased genes.

Relationship between transcriptional modules and bias-specific eigenvectors

We assessed similarity of the transcriptional modules inferred by Ayroles et al. (2009) with the sex-bias class PCs used as traits in the quantitative genetic analysis, producing the results shown in Table S4. While there are many nominally significant correlations, few of them are of substantial size. Overall, there is little tendency for the modules to line up clearly with the PC traits used in our analysis, reflecting the differences in the algorithms used. The two transcriptional modules identified as male-biased and female-biased by Ayroles et al.(2009) are particularly illuminating. Male biased module 7 had highly significant but very small correlations with the two male-biased PC traits, but was also significantly correlated with female-biased and relatively unbiased trait PCs. Female-biased module 18 likewise had one slightly higher correlation with a female-biased trait PC, but was also correlated with all the remaining trait PCs. The single apparent exception is module 50, which had a correlation of 0.62 with FB2, and low correlations with unbiased trait PCs. Overall, the pattern of genetic covariance among genes is structured quite differently from that of the modules recovered by Ayroles et al. (2009).

Literature Cited

Ayroles, J. F., M. A. Carbone, E. A. Stone, K. W. Jordan, R. F. Lyman, M. M. Magwire, S. M. Rollmann et al. 2009. Systems genetics of complex traits in Drosophila melanogaster. Nature Genetics 41:299-307.

Table S1. Pearson correlations of dimorphism with predictor variables.

	$\bar{E}$	τ	$\log_{10}(\bar{g})$	r_{mf}	$\bar{r}_w$	$\bar{r}_b$	$\log_{10}(\lvert m-f\rvert)$	$\lvert\bar{r}_{\delta w}\rvert$	$\log_{10}(\lvert\bar{r}_{\delta b}\rvert+0.01)$
$\log_{10}(\lvert D\rvert+0.01)$	-0.032	0.356	0.244	-0.414	0.185	0.146	0.342	0.231	0.097
$\bar{E}$		-0.268	-0.108	0.235	0.157	-0.007	-0.211	-0.182	-0.058
τ			0.556	-0.108	-0.231	0.130	0.462	0.273	0.343
$\log_{10}(\bar{g})$				0.265	-0.177	0.046	0.700	0.040	0.576
r_{mf}					-0.134	-0.96	-0.067	-0.260	0.228
$\bar{r}_w$						0.142	-0.146	-0.192	-0.143
$\bar{r}_b$							0.081	0.069	0.036
$\log_{10}(\lvert m-f\rvert)$								0.144	0.377
$\lvert\bar{r}_{\delta w}\rvert$									0.088

Note: All correlation coefficients are nominally significant at P<0.0001, except for $\bar{E}$ with $\log_{10}(\lvert D\rvert+0.01)$, for which P=0.001. The P-values are statistically invalid due to the lack of independence of the underlying expression data.

Table S2. Genetic variances and covariances (diagonal and below) and genetic correlations (above the diagonal) for all 8 principal component scores in both sexes.

Expression Sex	Trait	MB1	MB2	FB1	FB2	UB1	UB2	UB3	UB4	MB1	MB2	FB1	FB2	UB1	UB2	UB3	UB4
Male	MB1	**53.664**	-0.191	0.043	-0.053	-0.469	-0.542	-0.352	-0.050	**0.229**	0.070	-0.102	-0.021	0.089	-0.486	-0.150	-0.031
Male	MB2	-5.674	**16.539**	-0.268	-0.152	-0.180	-0.353	0.492	0.253	-0.082	**0.344**	-0.069	-0.320	-0.025	-0.427	0.509	0.076
Male	FB1	0.721	-2.515	**5.340**	0.536	-0.265	-0.065	-0.466	0.136	-0.674	-0.742	**0.614**	0.291	-0.378	-0.183	-0.499	0.313
Male	FB2	-0.210	-0.333	0.667	**0.290**	0.164	-0.151	-0.590	0.337	-0.217	-0.206	0.111	**0.644**	0.095	-0.118	-0.687	0.403
Male	UB1	-15.810	-3.370	-2.822	0.407	**21.182**	0.009	-0.146	-0.160	0.310	0.148	-0.484	-0.142	**0.601**	0.185	-0.145	-0.328
Male	UB2	-28.904	-10.437	-1.095	-0.592	0.297	**52.991**	0.046	-0.100	0.120	0.091	0.273	0.202	-0.385	**0.903**	-0.151	0.081
Male	UB3	-16.703	12.964	-6.983	-2.061	-4.343	2.188	**42.017**	0.097	-0.307	-0.045	-0.029	-0.288	-0.140	-0.013	**0.935**	-0.023
Male	UB4	-2.142	5.986	1.832	1.054	-4.286	-4.222	3.642	**33.798**	-0.337	0.009	0.087	-0.060	-0.026	-0.103	0.088	**0.855**
Female	MB1	4.115	-0.816	-3.819	-0.286	3.500	2.144	-4.876	-4.795	**6.006**	0.860	-0.576	-0.122	0.433	0.269	-0.210	-0.377
Female	MB2	0.856	2.348	-2.881	-0.187	1.141	1.108	-0.489	0.087	3.541	**2.825**	-0.592	-0.152	0.403	0.230	0.044	-0.051
Female	FB1	-7.028	-2.640	13.341	0.561	-20.944	18.657	-1.773	4.758	-13.277	-9.355	**88.456**	-0.010	-0.949	-0.126	-0.246	0.037
Female	FB2	-0.834	-7.205	3.717	1.919	-3.608	8.127	-10.339	-1.929	-1.660	-1.416	-0.530	**30.621**	0.038	0.301	-0.406	0.305
Female	UB1	6.590	-1.034	-8.831	0.519	27.955	-28.299	-9.185	-1.553	10.708	6.844	-90.163	2.150	**102.110**	0.027	0.089	0.014
Female	UB2	-20.326	-9.927	-2.421	-0.362	4.870	37.544	-0.480	-3.403	3.759	2.207	-6.759	9.510	1.554	**32.632**	-0.107	0.169
Female	UB3	-6.763	12.714	-7.085	-2.272	-4.097	-6.740	37.234	3.137	-3.156	0.450	-14.234	-13.809	5.511	-3.748	**37.720**	-0.005
Female	UB4	-1.422	1.939	4.528	1.360	-9.463	3.703	-0.912	31.123	-5.786	-0.536	2.202	10.566	0.885	6.028	-0.172	**39.215**

Table S3: Genetic variances and standard errors of variance estimates for principal component scores within data subsets defined by degree of sex bias in gene expression for the 16 trait data set.

			Male		Female		
Subset	PC	Code	Variance	S.E.	Variance	S.E.	*d*
Male Biased	1	MB1	53.66	14.83	6.00	4.26	0.60
Male Biased	2	MB2	16.54	4.30	2.82	1.96	0.71
Female biased	1	FB1	5.34	2.42	88.45	22.98	0.46
Female biased	2	FB2	0.29	0.12	30.62	7.31	0.19
Unbiased	1	UB1	21.18	4.93	102.11	23.60	0.75
Unbiased	2	UB2	52.99	12.25	32.63	7.97	0.97
Unbiased	3	UB3	42.02	9.93	37.72	9.13	1.00
Unbiased	4	UB4	33.80	8.24	39.22	9.62	1.00

Table S4. Vector correlations between the absolute value of the bias-class eigenvectors and the vector of module identities from Ayroles et al. (2009). Significance of correlations assessed relative to a null distribution of 1,000 vector correlations between module identities with random vectors.

		Bias-class Eigenvector							
module	genes	MB1	MB2	FB1	FB2	UB1	UB2	UB3	UB4
7	1319	0.112**	0.132**	0.072**	0.061**	0.033**	0.012	0.02*	0.023**
18	3505	0.073**	0.057**	0.101**	0.262**	0.081**	0.112**	0.073**	0.113**
23	114	0.054**	0.112**	0.033*	0.077**	0.012	0.005	0	0.004
34	85	0.007	0.018			0.002	0	0.009	0.032**
50	93			0.013	0.623**	0.027**	0.020**	0.043**	0.029**
88	106	0.006	0.015			0.001	0.013	0.008	0.038**
91	277	0.05	0.008	0.005	0.02	0.132**	0.079**	0.036**	0.036**
99	152	0.047**	0.005			0.071**	0.014*	0.006	0.007
112	51	0.003	0.017	0.005	0.006	0.009	0.011	0.008	0.016*
123	82					0.005	0.022**	0.020**	0.014
131	78	0.023	0.005			0.087**	0.014	0.047**	0.004
158	53	0.007	0.054*			0.011	0.004	0	0.008
169	55					0.027**	0.013	0.002	0.011
185	76			0.03	0.015	0.021**	0.039**	0.058**	0.075**
194	105			0.016	0.008	0.069**	0.015*	0.014	0.022**
211	113	0.008	0.013			0.031**	0.019**	0.002	0.003

Note: Analysis includes only transcriptional modules in which 50 or more genes with significant genetic variation were assigned in Ayroles et al. (2009); Blank cells are modules to which no gene in that sex-bias class was assigned.

* P<0.05

** P<0.01.

Table S5. Responses of dimorphism in relatively unbiased gene expression traits to selection on male- and female-biased expression.

Selection Regime[a]	UB $\|\Delta\|$ [b]
MB1	13.9 (4.3-27.4)
MB2	4.4 (1.8-8.8)
FB1	38.3 (21.2-59.1)
FB2	9.0 (4.5-15.8)
MB	10.7 (3.3-21.7)
FB	25.2 (13.5-40.0)
Bias	10.9 (3.4-21.8)

[a]MB1, MB2, FB1, FB2 directional selection the indicated trait; MB, FB directional selection on biased traits in one sex; Bias=directional selection on all sex-biased traits simultaneously.

[b]Total change in dimorphism for UB traits. Estimates are mean magnitude of change in dimorphism, (2.5% - 97.5% quantiles).

Table S6. Predicted conditional responses (medians and 2.5%-97.5% quantiles) of dimorphism of UB traits to antagonistic (A) or concordant (C) selection when other UB traits are held constant.

	Antagonistic[b]		Concordant[b]		UB $\|\Delta\|$ [c]		
Sel.[a]	*c*	*R*	*c*	*R*	A	C	ratio
UB1-c	8.7 (1.7-17.2)	15.2 (2.6-32.7)	36.2 (7.4-71.0)	38.7 (8.1-75.8)	6.2 (1.2-12.2)	8.8 (0.4-20.1)	0.69 (0.39-3.95)
UB2-c	2.7 (0.7-5.2)	4.4 (1.4-9.9)	35.0 (12.2-64.2)	35.2 (12.2-64.4)	1.9 (0.5-3.7)	2.3 (0.1-6.5)	0.80 (0.20-16.53)
UB3-c	1.6 (0.3-4.1)	3.6 (0.8-10.2)	57.6 (27.6-92.8)	57.8 (27.9-92.9)	1.2 (0.2-2.9)	2.1 (0.1-6.9)	0.51 (0.12-11.77)
UB4-c	1.9 (0.6-3.9)	3.2 (1.0-7.7	51.7 (20.7-86.1)	51.9 (20.9-86.3)	1.4 (0.4-2.8)	1.6 (0.1-5.0)	0.85 (0.21-17.53)

[a]Selection regime: the named trait is directionally selected, while the other UB traits are held constant.

[b]Directionally selected male traits have positive gradients, while directionally selected female traits negative selection gradients; *c*=conditional evolvability, the response in the direction of selection; *R*= respondability, the total response to selection including the MB and FB traits.

[c]Total change in dimorphism for UB traits under A or C selection; ratio=$\|\Delta_A\|/\|\Delta_C\|$.
